# The COVID-19 pandemic impact on prenatal anxiety

**DOI:** 10.1192/j.eurpsy.2022.1331

**Published:** 2022-09-01

**Authors:** K. Nourchene, E. Khelifa, O. Maatouk, S. Ben Aissa, B. Abassi, I. Bouguerra, B. Anissa, F. Amdouni, L. Mnif

**Affiliations:** 1 Razi hospital, Skolly, Tunis, Tunisia; 2 Razi, Skolly, Manouba, Tunisia; 3 Razi Hospital, F Adult Psychiatry Department, Manouba, Tunisia; 4 Razi Hospital, Psychiatry Ibn Omran, Manouba, Tunisia; 5 Errazi hospital-Mannouba, F, Ben Arous, Tunisia; 6 Mongi slim, Gynecology, tunis, Tunisia

**Keywords:** Pregnancy, Anxiety, Covid-19

## Abstract

**Introduction:**

Anxiety manifestations are one of the most described symptoms during pregnancy. Meanwhile, the effect of the coronavirus disease 2019 (COVID-19) pandemic on the mental health and anxiety distress in particular, of pregnant and postpartum women remains unclear.

**Objectives:**

The purpose of our study was to evaluate anxiety among prgnant women during covid19 and describe its associated factors

**Methods:**

It was a comparative cross-sectional case- control study in a Tunisian gynecologic department. All women were in the third term of pregnancy. Anxiety symptoms were evaluated using Beck Anxiety Inventory (BAI). The data were compared to a control group assessed in a similar study conducted before the pandemic in the same city. Eighty pregnant women was investigated during the covid pandemic and 100 pregnant women investigated before the COVID-19 outbreak in Tunisia was assigned to the control group.

**Results:**

Pregnant women during COVID-19 scored less on BAI than controls (15.49±9.223 vs 17.40±7.410). Less patients presented moderate to severe anxiety during pandemic (38.8% (n=31) than controls 51% (n=51)). The difference between groups in means and prevalence values was not significant. The negative results could be related to the low power of the test (P=0.36).

**Conclusions:**

Despite the expected psychological distress among vulnerable population , Covid-19 didn’t impact anxiety prevalence or scores among pregnant women in our current study .

**Disclosure:**

No significant relationships.

